# Electrical bioimpedance in the era of artificial intelligence

**DOI:** 10.2478/joeb-2024-0001

**Published:** 2024-01-26

**Authors:** Jie Hou, Naimahmed Nesaragi, Christian Tronstad

**Affiliations:** 1 Department of Physics, University of Oslo, 0316 Oslo, Norway; 2 The Intervention Centre, Oslo University Hospital, 0372 Oslo, Norway; 3 Department of Clinical and Biomedical Engineering, Oslo University Hospital, 0372 Oslo, Norway

Today, the first thing that comes to mind when someone mentions bioimpedance might be body composition estimation, and when someone mentions AI, we might immediately think of chatbots. The progression of AI over the preceding decades has witnessed a transition from attempting to artificially replicate neural communication in the human brain during the 1950s to contemporary emphasis on ethical considerations associated with the responsible utilization of AI in the 2020s.

The bandwagon of AI has influenced many fields of science. Given that electrical bioimpedance is often a multi-variable measurement for the prediction of a physiological state, the developments in AI-based solutions are certainly relevant to our field as well. An integration of electrical bioimpedance and AI could be important in the development of future health monitoring devices, driven by a shift from reactive treatment (after symptom onset) to preventive self-care. From simple neural networks to deep learning (DL), machine learning (ML) has already been used for over a decade in the development of prediction models based on bioimpedance data. More recently it has been used to improve several applications of electrical bioimpedance such as cuffless blood pressure [1, 2, 3], body composition analysis [4], non-invasive blood glucose measurement [5], classification of spectroscopic data (i.e., electrical impedance spectroscopy (EIS)) [6, 7] and electrical impedance tomography (EIT) [8, 9, 10].

Consider the non-invasive imaging technique EIT as an example, it reconstructs the spatial distribution of the passive electrical properties of the sensing area, relying on data processing and reconstruction algorithms. Recent substantial progress in leveraging DL techniques for AI-based medical imaging has prompted considerable interest and attention in applying DL to EIT-based image reconstruction. The comprehensive overview of such advancements made in this direction spans three key avenues: single network reconstruction, synergistic integration of DL with traditional algorithmic EIT reconstruction, and the fusion of multiple networks for hybrid reconstruction [9, 10, 11]. Further, as the field of AI progresses rapidly, the recently introduced deep image prior, a variant of convolutional neural network, can be employed to improve a given EIT image without requiring any pre-existing training data. This technique may surpass the state-of-the-art EIT reconstruction methodologies [12].

Although AI-based tools offer great possibilities for improving the performance of bioimpedance technology, there are some challenges that are more or less specific to our field. In general, effective training of robust AI-based applications necessitates a substantial volume of data, a process inherently time-consuming and labor-intensive. This challenge is particularly pronounced in medical applications, where the acquisition of human-centric data is notably challenging.

As AI training requires large volumes of data, and bioimpedance data is often limited in size, one solution can be to collect and combine data from different studies and laboratories. However, several factors can prevent researchers from doing so. Specifically for bioimpedance, factors such as electrode type, size, placement and impedance analyzers used could influence the measured impedance largely. One way to tackle this problem is to have both open-source databases as well as open-source tools for electrical impedance data where it is possible to integrate data from for example different instruments and electrodes [13]. Another way to increase data volume and assist in AI training is by generating synthetic data, an approach that holds several potential benefits. Synthetically generating bioimpedance data would give us the possibility to evaluate AI ideas at an early stage, without being influenced by the bias introduced by experimental and instrumental variations. Several works in other medical fields have proposed the use of the generative adversarial network (GAN) to generate synthetic data, both to enlarge the database for AI models in a controlled way and to reduce the use of animals for experiments [14, 15, 16]. New possibilities in the generation of synthetic 3D medical images may translate to easier access to digital human phantoms for bioimpedance research in the future [17, 18].

With increasing complexity of ML models, the interpretability of the models poses growing challenges. In addressing this issue, there has been a rise in the adoption of explainable artificial intelligence (XAI) in recent years. One precondition to be able to employ AI-based models in real-life applications is to be able to use them responsibly, which involves the ability to trust the model’s output. This trust, in turn, demands a significant level of interpretability. Nevertheless, having explainable models does not always make the models understandable. Compared to other types of variables in prediction models, such as blood lipids or ECG features, electrical bioimpedance is generally not a measurement technique that patients or clinicians are familiar with. Therefore, it may be difficult to understand the predictions even when XAI is used. Consequently, XAI may not always provide full interpretability for users unless additional explanation is provided.

Applications of AI in electrical bioimpedance are not only limited to data analysis and prediction, but may also improve the measurement. Artifacts are not uncommon in electrical bioimpedance measurements, particularly in scenarios like ambulatory monitoring over time, where AI-based solutions may help to identify and remove artifacts before further data analysis [19]. Another example is the optimization of electrode positions in 2D EIT with the aid of a deep learning approach [20]. Further, EIT image reconstruction owes to inherent nonlinearity and ill-posedness, presenting challenges for classical regularization techniques. Deep generative models such as variational autoencoder networks, normalizing flow, and score-based diffusion models have been shown to play a crucial role in learning implicit regularizers and prior knowledge for EIT-based reconstruction tasks [21].

In the future, we will likely see new ways AI can assist theoretical, experimental and applied areas of electrical bioimpedance that are currently unforeseen.

## References

[1] Ibrahim B, Jafari R. (2022). Cuffless Blood Pressure Monitoring from a Wristband with Calibration-Free Algorithms for Sensing Location Based on Bio-Impedance Sensor Array and Autoencoder. Sci Rep.

[2] Sel K, Osman D, Huerta N, Edgar A, Pettigrew RI, Jafari R. (2023). Continuous Cuffless Blood Pressure Monitoring with a Wearable Ring Bioimpedance Device. npj Digit. Med.

[3] Kireev D, Sel K, Ibrahim B, Kumar N, Akbari A, Jafari R, Akinwande D. (2022). Continuous Cuffless Monitoring of Arterial Blood Pressure via Graphene Bioimpedance Tattoos. Nat. Nanotechnol.

[4] Nematollahi MA, Askarinejad A, Asadollahi A, Bazrafshan M, Sarejloo S, Moghadami M, Sasannia S, Farjam M, Homayounfar R, Pezeshki B, Amini M, Roshanzamir M, Alizadehsani R, Bazrafshan H, Bazrafshan drissi H, Tan RS, Acharya UR, Islam MSS. (2023). A Cohort Study on the Predictive Capability of Body Composition for Diabetes Mellitus Using Machine Learning. J Diabetes Metab Disord.

[5] Sanai F, Sahid AS, Huvanandana J, Spoa S, Boyle LH, Hribar J, Wang DTY, Kwan B, Colagiuri S, Cox SJ, Telfer TJ. (2023). Evaluation of a Continuous Blood Glucose Monitor: A Novel and Non-Invasive Wearable Using Bioimpedance Technology. J Diabetes Sci Technol.

[6] Pandeya SR, Nagy JA, Riveros D, Semple C, Taylor RS, Hu A, Sanchez B, Rutkove SB. (2022). Using Machine Learning Algorithms to Enhance the Diagnostic Performance of Electrical Impedance Myography. Muscle & Nerve.

[7] Schaeffer J, Gasper P, Garcia-Tamayo E, Gasper R, Adachi M, Gaviria-Cardona JP, Montoya-Bedoya S, Bhutani A, Schiek A, Goodall R, Findeisen R, Braatz RD, Engelke S. (2023). Machine Learning Benchmarks for the Classification of Equivalent Circuit Models from Electrochemical Impedance Spectra. J. Electrochem. Soc.

[8] Chen X, Wang Z, Zhang X, Fu R, Wang D, Zhang M, Wang H. (2021). Deep Autoencoder Imaging Method for Electrical Impedance Tomography. IEEE Transactions on Instrumentation and Measurement.

[9] Wu Y, Chen B, Liu K, Zhu C, Pan H, Jia J, Wu H, Yao J. (2021). Shape Reconstruction With Multiphase Conductivity for Electrical Impedance Tomography Using Improved Convolutional Neural Network Method. IEEE Sensors Journal.

[10] Ren S, Guan R, Liang G, Dong F. (2021). RCRC: A Deep Neural Network for Dynamic Image Reconstruction of Electrical Impedance Tomography. IEEE Transactions on Instrumentation and Measurement.

[11] Ren S, Sun K, Tan C, Dong F. (2020). A Two-Stage Deep Learning Method for Robust Shape Reconstruction With Electrical Impedance Tomography. IEEE Transactions on Instrumentation and Measurement.

[12] Liu D, Wang J, Shan Q, Smyl D, Deng J, Du J. (2023). DeepEIT: Deep Image Prior Enabled Electrical Impedance Tomography. IEEE Transactions on Pattern Analysis and Machine Intelligence.

[13] Murbach MD, Schwartz DT. (2019). Open Software and Datasets for the Analysis of Electrochemical Impedance Spectra. Electrochem. Soc. Interface.

[14] Chen X, Roberts R, Liu Z, Tong W. (2023). A Generative Adversarial Network Model Alternative to Animal Studies for Clinical Pathology Assessment. Nat Commun.

[15] Guttulsrud H. (2023). Generating Synthetic Medical Images with 3D GANs.

[16] Mensing D, Hirsch J, Wenzel M, Günther M. (2022). 3D (c)GAN for Whole Body MR Synthesis. Deep Generative Models: Second MICCAI Workshop, DGM4MICCAI 2022, Held in Conjunction with MICCAI 2022, Singapore, September 22, 2022, Proceedings.

[17] Schaefferkoetter J, Yan J, Moon S, Chan R, Ortega C, Metser U, Berlin A, Veit-Haibach P. (2021). Deep Learning for Whole-Body Medical Image Generation. Eur J Nucl Med Mol Imaging.

[18] Wu C, Zhang H, Chen J, Gao Z, Zhang P, Muhammad K, Del Ser J. (2022). Vessel-GAN: Angiographic Reconstructions from Myocardial CT Perfusion with Explainable Generative Adversarial Networks. Future Generation Computer Systems.

[19] Moeyersons J, Morales J, Seeuws N, Van Hoof C, Hermeling E, Groenendaal W, Willems R, Van Huffel S, Varon C. (2021). Artefact Detection in Impedance Pneumography Signals: A Machine Learning Approach. Sensors.

[20] Smyl D, Liu D. (2020). Optimizing Electrode Positions in 2-D Electrical Impedance Tomography Using Deep Learning. IEEE Transactions on Instrumentation and Measurement.

[21] Wang H, Xu G, Zhou Q. (2024). A Comparative Study of Variational Autoencoders, Normalizing Flows, and Score-Based Diffusion Models for Electrical Impedance Tomography. Journal of Inverse and Ill-posed Problems.

